# Crystal structure refinement with *SHELXL*

**DOI:** 10.1107/S2053229614024218

**Published:** 2015-01-01

**Authors:** George M. Sheldrick

**Affiliations:** a Department of Structural Chemistry, Georg-August Universität Göttingen, Tammannstraße 4, Göttingen 37077, Germany

**Keywords:** *SHELXL*, crystal structure refinement, X-ray and neutron diffraction, SHREDCIF

## Abstract

The improvements in the crystal structure refinement program *SHELXL* have been closely coupled with the development and increasing importance of the CIF (Crystallographic Information Framework) format for validating and archiving crystal structures. An important simplification is that now only one file in CIF format (for convenience, referred to simply as ‘a CIF’) containing embedded reflection data and *SHELXL* instructions is needed for a complete structure archive; the program *SHREDCIF* can be used to extract the .hkl and .ins files required for further refinement with *SHELXL*. Recent developments in *SHELXL* facilitate refinement against neutron diffraction data, the treatment of H atoms, the determination of absolute structure, the input of partial structure factors and the refinement of twinned and disordered structures. *SHELXL* is available free to academics for the Windows, Linux and Mac OS X operating systems, and is particularly suitable for multiple-core processors.

## Abstract 2

New features added to the refinement program *SHELXL* since 2008 are described and explained.

## Introduction   

The first version of *SHELX* dates back to about 1970 and, after extensive testing, it was first released in 1976. Since then the program system has been developed continuously. The early history has been described by Sheldrick (2008 ▶). The present paper is intended to explain the philosophical and crystallographic background to developments between 2008 and 2015 in *SHELXL*, the program in the *SHELX* system responsible for crystal structure refinement. Although *SHELXL* may also be used for the refinement of macromolecular structures against high-resolution data, most of the new developments have concentrated on the refinement of chemical structures, such as those published in *Section C* of *Acta Crystallographica*. Readers not familiar with *SHELX* may find it useful to look at Sheldrick (2008 ▶) before reading this paper.

A major change since 2008 is that the distribution is performed *via* the *SHELX* homepage (http://shelx.uni-ac.gwdg.de/SHELX/), which also provides a great deal of documentation, tutorials and other useful information. The programs are updated more frequently than in the past and the list of recent changes should be consulted regularly to see if it is necessary to download a new version. The homepage also contains a list of registered users (but not their email addresses); currently there are over 8000 spread over more than 80 countries. *SHELX* workshops are announced on the homepage, and many of the talks given at these workshops may be downloaded there. *SHELXL* is compiled with the Intel ifort FORTRAN compiler using the statically linked MKL library, and is available free to academics for the 32- or 64-bit Windows, 32- or 64-bit Linux and 64-bit Mac OS X operating systems. Multithreading is achieved using *OpenMP* along the lines suggested by Diederichs (2000 ▶), and the program is particularly suitable for multiple-core processors.

## *SHELXL* and CIF format   

### The importance of depositing crystallographic data   

Although the IUCr journals have led the way in insisting that experimental crystallographic data should be deposited, several leading chemical journals still only require the deposition of a CIF (Hall *et al.*, 1991 ▶) containing just the results of the crystal structure determination and not the X-ray or neutron reflection data used to determine the structure. In this respect, biological crystallographers are more advanced. The PDB (Protein Data Bank; Berman, 2008 ▶) has required the deposition of reflection data since February 2008 and virtually all journals that report biological crystal structures, including high-profile journals such as *Nature* and *Science,* require a PDB ID for the structure. This has already had a considerable impact. For example, it has led to the retraction of several structures in which the data do not support the claim that a particular ligand was bound to a protein.

One very recent example of the use of such deposited data (Köpfer *et al.*, 2014 ▶) can be mentioned here, since it involved the use of *SHELXL* to refine occupancies and obtain standard uncertainties for them. For over 50 years, the accepted model (Hodgkin & Keynes, 1955 ▶) for the potassium channel present in many living systems was that it involved the transport of both potassium ions and water molecules, based on the argument that adjacent binding sites could not be occupied by K^+^ cations because they would repel one another, and so the intermediate sites must be occupied by water molecules. Several protein crystal structures were refined at modest resolution with alternating potassium ions and water molecules in the channel and appeared to support this model. However, to the authors’ credit, they deposited their reflection data, including the Friedel pairs, although that was not then obligatory. When sophisticated molecular dynamics (MD) calculations showed that only a model with adjacent K^+^ cations could account, by a sort of knock-on effect, for the very high potassium permeability observed, it was necessary to reinvestigate the structure using the deposited X-ray reflection data. Both the occupancy refinements with *SHELXL* and the analysis of the anomalous data with *SHELXD* (Schneider & Sheldrick, 2002 ▶) and *ANODE* (Thorn & Sheldrick, 2011 ▶) showed conclusively that the four connected potassium sites are almost fully occupied, as predicted by the MD calculations.

### Archiving crystallographic data   

To make the deposition and archiving of reflection data as simple as possible, the CIF written by *SHELXL* now includes the .hkl reflection data file, embedded as CIF text:

_shelx_hkl_file

;

... reflection data in SHELX HKLF 2, 3, 4 or 5 format ...

;

_shelx_hkl_checksum 12345

The checksum provides a check that the data have not been corrupted accidentally. The .res results file from the refinement and the .fab file (see below), if used in the refinement, are embedded into the CIF in the same way. The *SHELX* program *SHREDCIF* may be used to extract these files from the CIF archive and rename the .res file to .ins, for example to perform further refinements with *SHELXL*. The intention is that such CIFs containing embedded data should become standard for deposition and archiving. It is particularly convenient that only one file is needed. CIF identifiers beginning with _shelx_ are reserved for use by the *SHELX* programs, but of course other program authors may use a similar construction for embedding the reflection data *etc*. Users who do not wish to preserve their carefully measured data for posterity in this way have criticized the embedding of the reflection data on the grounds that (*a*) the resulting CIF is too large for submission with a paper for publication and that (*b*) certain CIF-processing programs take a long time to read such a CIF and may even choke in the attempt. However, it should be noted that (*a*) the figures submitted with a paper often involve larger files and (*b*) *SHREDCIF* can usually read and dismember such a CIF in less than one second! To generate a CIF without intensity data for other purposes, *e.g.* for input to a molecular graphics program, the keyword NOHKL may be used in the *SHELXL* ACTA instruction.

It is difficult to understand why several leading chemical journals still only require the deposition of the atom co­ordinates, *etc.,* but not the reflection data, especially now that the Cambridge Structural Database (CSD; Allen, 2002 ▶) accepts the new CIFs and strongly encourages deposition of the reflection data. A simple solution would be for journals to require a confirmation that the full data have been deposited with the CSD (Bruno & Groom, 2014 ▶) or COD (Gražulis *et al.*, 2012 ▶), analogous to the way in which the PDB requires deposition of the structural and reflection data before issuing a PDB ID.

### Including CIF items at the end of the .hkl file   

Since *SHELX76*, the reflection data have been read until a reflection with indices 0,0,0 or a blank line (or card) or the end of the file was encountered. The rest of the file was never read by the *SHELX* programs. This means that additional data specific to that data set, such as details of the data collection and processing, may conveniently be appended to the .hkl file, which is a much safer way of preserving them than putting them in a separate file. For example, the Bruker scaling program *SADABS* (Krause *et al.*, 2015 ▶) now appends CIF format items such as those shown below to the .hkl file that it outputs:

_exptl_absorpt_process_details ‘SADABS 2014/4’

_exptl_absorpt_correction_type multi-scan

_exptl_absorpt_correction_T_max 0.7489

_exptl_absorpt_correction_T_min 0.7208

_exptl_special_details

;

The following wavelength and cell were deduced by SADABS from the direction cosines etc. They are given here for emergency use only:

CELL 0.71072 6.100 18.294 20.604 90.006 89.992 90.000

;

*SHELXL* uses the CIF items found at the end of the .hkl file to replace items to which it would otherwise have given the value ‘?’. It ignores all other items. So in this example, the first four CIF items find their way (left justified) into the output CIF, but although _exptl_special_details is legal for a CIF it is not included as a CIF item because this CIF identifier would not otherwise have been output. However, it is still included in the .cif file as part of the embedded .hkl file, so that the information is not lost. Unfortunately, because of a fundamental CIF design weakness (the same character ‘;’ is used for both the beginning and end of a text item; it would have been better to have used a different terminator such as ‘:’), *SHELXL* has to replace ‘;’ in this example by ‘)’ when embedding the .hkl file, and *SHREDCIF* repairs the damage by turning a leading ‘)’ in an otherwise blank line back to ‘;’. In this example, the cell following _exptl_special_details is not the same as in the CELL instruction used in the .ins file, because there is a reorientation matrix in the HKLF 4 instruction to transform the indices to the conventional *P*2_1_2_1_2 setting for the space group. However, it is still useful to preserve it in case the .hkl file becomes orphaned.

## Refinement against neutron diffraction data and special facilities for H atoms   

The new features in *SHELXL* for refinement against neutron data have been discussed recently by Gruene *et al.* (2014 ▶). If a NEUT instruction is placed before SFAC, neutron scattering factors are employed, and the default bond lengths to H or D atoms are lengthened to correspond to internuclear distances rather than the distances appropriate for refinement against X-ray data. Whereas for X-rays H and D are treated specially, for neutrons they are treated as normal atoms. The HFIX and AFIX instructions may still be used to generate starting positions for H and D atoms, but it is recommended to use geometric restraints rather than a riding model for their refinement against neutron data. This is particularly important when anti-bumping restraints are applied; they work much better for a restrained than for a riding-model refinement of the H and D atoms against neutron data.

### Chiral volume restraints for refinement against neutron data   

The chiral volume restraint CHIV, which is often used for macromolecular refinement, is interpreted as follows if NEUT is set. If three atoms other than H or D are bonded to the atom in question, the H and D atoms are ignored and the CHIV restraint operates in the same way as for a refinement against X-ray data. If there are exactly three bonded atoms including H and D, the latter are used in the restraint. Thus, CHIV 0 N1 could be used to restrain a terminal –NH_2_ group to be planar, and CHIV with a nonzero target value could be used to make it nonplanar.

### Anisotropic refinement of H and D atoms against neutron data   

Since the neutron scattering factors for H, and especially for D, are of a similar order of magnitude to those for other atoms, H and D also need to be refined anisotropically for refinement against neutron data. Unfortunately, this results in about twice as many parameters as for a standard refinement against X-ray data, and the number of data available may well be less than for an X-ray refinement, so further restraints such as the new RIGU rigid-bond restraint (Thorn *et al.*, 2012 ▶) may be required.

The RIGU restraints require that the relative motion of bonded atoms is at right angles to the bond joining them. This sets up three restraints per atom pair, one of which is equivalent to the classical rigid-bond restraint DELU. RIGU is very generally applicable and it is almost always safe to add a RIGU instruction without further parameters to the .ins file. The resulting displacement ellipsoid plots tend to appear chemically more reasonable than those from an unrestrained refinement and there is usually little change in the final *R* factors.

The following example, using data from Lübben *et al.* (2014 ▶), is a little different, because it involves the anisotropic refinement of all atoms, including H atoms, using *SHELXL* against neutron diffraction data collected at 9 K. The .ins file was the same as that used for refinement against X-ray data, except that: (i) a NEUT instruction was placed before SFAC, so that neutron scattering lengths were used instead of X-ray scattering factors; (ii) instead of using a riding model for the refinement of the H atoms, SADI (equal distance) restraints were applied to the O—H bonds in the water molecule, the C—H bonds in the CH_2_ and CH_3_ groups, and the H⋯H distances within the CH_3_ group; and (iii) a much larger value was obtained for the extinction parameter (EXTI).

**Figure 1:**
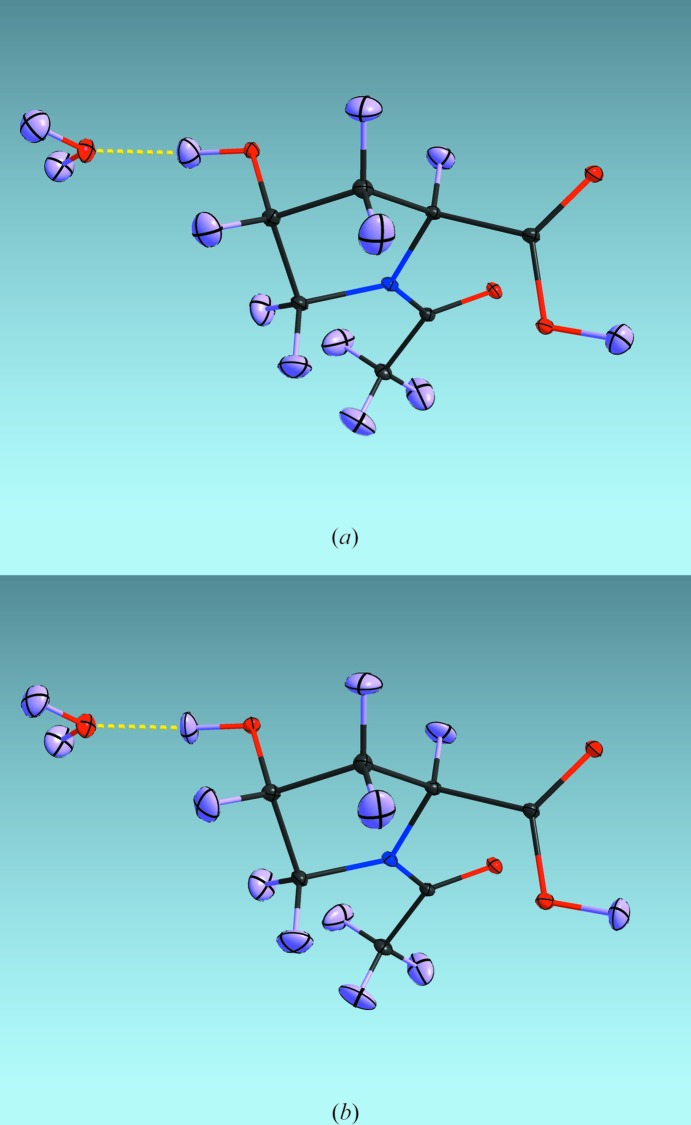
Displacement ellipsoid style plot, drawn using *SHELXLE* (Hübschle *et al.*, 2011 ▶), of *N*-acetyl-4-hydroxy-l-proline monohydrate, (*a*) without and (*b*) with the application of RIGU restraints to the anisotropic displacement parameters. Both the orientations of the 50% probability displacement ellipsoids and the *R*_1_ values are very similar in the two cases, justifying the use of these restraints for the anisotropic refinement of H atoms against neutron data. When RIGU restraints are applied, the smallest principal component of the anisotropic displacement ellipsoid is aligned even more closely with the bond direction. Note that the common assumption that the isotropic *U* values of the H atoms can be set to 1.2 or 1.5 times the equivalent isotropic *U* values of the atoms to which they are attached would be most inappropriate for this structure determined at 9 K.

Close inspection of the atomic displacement ellipsoids in Fig. 1 ▶(*a*) shows that the assumption that the relative motion of the H atoms is at right angles to the bonds holds well, even for the unrestrained refinement. The refinement with tight RIGU restraints (RIGU 0.0001) for the bonded atoms (Fig. 1 ▶ *b*) looks very similar, but the H-atom displacement ellipsoids are aligned so that their smallest principal axes are even closer to the bond directions, as required when the motion is at right angles to the bonds. However, Fig. 1 ▶(*b*) also reveals a small weakness of the rigid-bond assumption: the H-atom displace­ment ellipsoids appear to be slightly squashed in the direction of the bond. This is probably because the amplitude of the zero-point motion along the bond is larger for the H atom than for the atom to which it is bonded, because of the smaller mass of the former, but the rigid-bond restraint tries to make them equal. As a result, the *R*_1_ value is slightly higher for the RIGU-restrained refinement (0.0342 rather than 0.0304). This effect is only observable here because of the extremely low temperature (9 K) and the high-quality data; at higher temperatures, the RIGU restraints can be very useful to stabilize the anisotropic refinement of H and other atoms against neutron data.

Fig. 1 ▶ also exhibits much larger atomic displacement ellipsoids for the H atoms than for the remaining atoms. At such low temperatures, the frequently made assumption that the isotropic displacement parameters of the atoms can be set to 1.2 or 1.5 times the equivalent isotropic *U* values of the atoms to which they are bonded is clearly not justified. However, at temperatures above about 100 K it has been shown that this assumption is less seriously flawed (Lübben *et al.*, 2014 ▶). Capelli *et al.* (2014 ▶) recently showed that Hirshfeld atom refinement provides a much more accurate way of deriving anisotropic displacement parameters for H atoms from X-ray data.

### Other new facilities for H atoms and CF_3_ groups   

Except where the NEUT instruction is used, both H and D are now treated as special in the input syntax. This is useful when both are present, *e.g.* when the crystals came from an NMR tube containing a deuterated solvent. The AFIX instructions for CH_3_ groups may now also be used to set up CF_3_ groups, but it is better to refine these as rigid groups or with distance restraints (DFIX or SADI) than by applying a riding model, because the latter can be unstable.

An HTAB instruction without any parameters instructs the program to find possible hydrogen bonds. These now include C—H⋯O interactions when the C atom is directly or in­directly attached to an electronegative atom (Taylor & Kennard, 1982 ▶). Such weak interactions involving H atoms attached to peptide C_α_ atoms are common in protein structures (Desiraju & Steiner, 1999 ▶). The resulting full HTAB and EQIV instructions are appended after the END instruction of the .res file and need to be (selectively) transferred to the beginning of the .ins file, so that they will be included in the CIF generated by the next refinement. This facilitates the generation of tables of hydrogen bonds, and helps to prevent hydrogen bonds involving symmetry-equivalent atoms from being overlooked.

## Absolute structure determination   

In the distant past, it was often assumed that it was necessary to include a heavy atom, *e.g.* by making a rubidium salt or bromobenzoate derivative, in order to obtain a reliable absolute structure, for instance to establish which enantiomer of a chiral molecule was correct. Since then, experimental and computational methods have made such progress that the absolute structure can often even be determined with Mo *K*α radiation when the heaviest atom is oxygen (Escudero-Adán *et al.*, 2014 ▶). When the 2008 *SHELX* paper was written, the method of choice to determine the absolute structure was to refine the Flack parameter (Flack, 1983 ▶) as one of the parameters in a full-matrix refinement. Since then it has become clear that this led to a substantial overestimation of the standard uncertainty of the Flack parameter, and that post-refinement methods using either a Bayesian approach (Hooft *et al.*, 2008 ▶) or quotients or differences of the Friedel opposites as observations (Parsons *et al.*, 2013 ▶) give more reasonable estimates of the Flack parameter, and especially its standard uncertainty. This led to the IUCr/*checkCIF* requirement that Friedel opposites should not be merged in the deposited data. For small-molecule refinements with *SHELXL*, the input .hkl file should contain the unmerged data. This enables the program to produce a more complete output CIF and to estimate the Flack parameter using the Parsons quotient method for all noncentrosymmetric structures. This approach works well even for twinned structures. For structure refinement, the reflections are, by default, merged according to the point group of the crystal structure (MERG 2 in *SHELXL* notation). In the relatively rare cases that result in an intermediate value of the Flack parameter with a small standard uncertainty, in order to obtain the most accurate calculated intensities and hence difference density, it is still necessary to refine the Flack parameter by the full-matrix method (TWIN/BASF). However, a Flack parameter of 0.5 with a small standard uncertainty is a warning sign that the true space group might be centrosymmetric!

## Estimates of standard uncertainties   

One side effect of the inclusion of Friedel opposites is that there will be nearly twice as many data for the refinement of a noncentrosymmetric structure, which, using the usual least-squares algebra, would lead to a reduction in the estimated standard uncertainties of all parameters by a factor of nearly 2^1/2^. *SHELXL* now uses the number of unique reflections as defined by the Laue group, rather than the number of observations, in the formula used to estimate the standard uncertainties (Spek, 2012 ▶). It could be argued that all reflection intensities are independent measurements, and this was approximately true for unscaled data from point detectors before the introduction of focusing optics. However, it is now standard practice to scale the data so that equivalent reflections (usually including Friedel opposites) become more equal, in order to correct for absorption and differences in the effective crystal volume irradiated, and then the equivalent reflections can no longer be regarded as independent observations. In some cases, this change may result in a modest increase in the estimated standard uncertainties, but these were generally underestimated anyway (Taylor & Kennard, 1986 ▶). The new method of estimating standard uncertainties also applies to twinned structures, where some *SHELXL97* users were required by referees to throw away some of their carefully measured data so that the number of observations would be equal to the number of unique reflections. Now all the experimental data may be used and the estimated standard uncertainties should be more realistic. With *SHELXL97*, it was necessary to use the third least-squares parameter to correct the estimated standard uncertainties; this is not required anymore (except for ‘SQUEEZEd’ structures).

## Input of partial structure factors   

The new ABIN instruction was primarily designed to facilitate the use of the SQUEEZE facility (Spek, 2015 ▶) in the program *PLATON* (Spek, 2009 ▶), but it can also be used to input a bulk solvent model for a macromolecule. *PLATON* calculates the partial structure factors corresponding to a blob of un­modelled difference density and writes them to the .fab file. The ABIN instruction causes *h*, *k*, *l*, *A* and *B* to be read from the .fab file, where *A* and *B* are the real and imaginary components, respectively, of a partial structure factor. These reflections are read in free format (one reflection per line) and may be in any order. Duplicates, systematic absences and reflections outside the resolution limits for refinement are ignored. Symmetry equivalents are generated automatically. At least one symmetry equivalent (according to the point group) of each reflection present in the .hkl file, including all reflections in all twin components if the structure is twinned, should be present in the .fab file. For twinned structures, it is necessary first to use the new LIST 8 instruction (see below) to generate detwinned data for input to *PLATON*. The *A* and *B* values refer to the untwinned structure, but in the case of a twinned structure, after applying the appropriate symmetry trans­formations, they are added to the calculated structure factors for all twin components.

ABIN takes two free variable numbers (Sheldrick, 2008 ▶) *n*_1_ and *n*_2_ as parameters. The *A* and *B* values read from the .fab file are multiplied by *k*exp[−8π^2^ *U*sin^2^θ/λ^2^], where *k* is the value of free variable *n*_1_ and *U* is the value of free variable *n*_2_. These two optional parameters may be needed when the partial structure factors come from a bulk solvent model of a macromolecule, but are probably not needed for use with SQUEEZE. SQUEEZE should only be used where it is not possible to model the disordered solvent by normal methods, *e.g.* when there is a continuous ribbon of diffuse difference density along one of the unit-cell axes. Partial structure factors and ABIN should always be used in preference to the old procedure of modifying the input .hkl file, which made it impossible to remodel the disordered density should a better method become available.

## Extending the PART number concept   

The use of PART numbers, introduced in *SHELXL93*, has proved invaluable in the refinement of disordered structures. Two atoms are considered to be bonded if they have the same PART number or if one of them is in PART 0. The resulting connectivity table is used for the generation of H atoms (HFIX and AFIX), for setting up restraints such as DELU, SIMU, RIGU, CHIV, BUMP and SAME, and for generating tables of geometric parameters (BOND, CONF, HTAB). Usually, most of the atoms are in PART 0, but, for example, a molecule or side chain dis­ordered over three positions could use PART 1, PART 2 and PART 3. If the PART number is negative, bonds are not generated to symmetry-equivalent atoms. It should be noted that positive PART numbers 1, 2, 3 *etc.* correspond to the alternative location indicators *A*, *B*, *C* *etc.* in PDB format. However, this notation is difficult to use when there is a disorder within a disorder. A BIND instruction that specifies two numbers may now be used to get around this problem. For example, BIND 2 4 means that, in addition to the usual PART rules, atoms in PART 2 may also bond to atoms in PART 4. Negative PART numbers are allowed in the BIND instruction.

As an example, consider an *n*-butyl substituent coordinated through atom C1 that splits into two disorder components at C2. Atom C1 is then in PART 0, C2*A*, C3*A* and C4*A* in PART 1, and C2*B*, C3*B* and C4*B* in PART 2. Atom C1 is bonded to both C2*A* and C2*B* but, because these two atoms have different PART numbers, H atoms will be generated correctly using the HFIX instruction. However, if there is a further disorder starting at atom C3*B*, this cannot be handled easily by *SHELX97*. Atoms C3*B* and C4*B* can be split into C3*B*′ and C4*B*′ in PART 3 and into C3*B*′′ and C4*B*′′ in PART 4, but then atoms C3*B*′ and C3*B*′′ are not bonded to C2*B* because they have different nonzero PART numbers. Extra bonds could have been inserted into the connectivity table with:

BIND C2B C3B’

BIND C2B C3B”

but then HFIX or AFIX would still not generate the correct H atoms, because they need to refer to the PART numbers of the neighbouring atoms too. However, the alternative

BIND 2 3

BIND 2 4

now enables the H atoms to be generated correctly. Since *SHELXL* allows atoms to have the same names if they have different PART numbers, atoms C3*A*, C3*B*′ and C3*B*′′ could all be labelled C3 in this example. This would simplify the naming of the H atoms, but might confuse non-*SHELX* programs that read the .res file. As with almost every disorder, the use of RIGU is strongly recommended here.

## Other new features in *SHELXL*   

One of the most common cases of instability in crystal structure refinements is when the atomic displacement parameters refine to appreciably negative values. The new XNPD instruction may be used to combat this. When an isotropic displace­ment parameter, or a principal component of an anisotropic displacement parameter, refines to a value less than (*e.g.* more negative) the value specified with the XNPD instruction, it is replaced by that value, and the displacement parameters *U*_iso_ or *U* ^*ij*^ are recalculated. Thus, the default setting of XNPD -0.001 avoids the risk of the refinement becoming unstable, but still leads to nonpositive definite (NPD) atoms being recognized and reported. For problematic cases, it may be desirable to set XNPD to a small positive value. However, it should then first be checked that the negative value was not caused by an error in the input file, *e.g.* an incorrect element type or site-occupation factor.

The new LIST 8 option writes *h*, *k*, *l*, *F*_o_^2^, σ(*F*_o_^2^), *F*_c_^2^, ϕ (phase angle in degrees), *d* spacing in ångström (Å) and 1/(*w* ^1/2^) in CIF format to the .fcf file, where *w* is the weight derived from the weighting scheme and used in the refinement. For weak reflections, 1/(*w* ^1/2^) should be only a little larger than σ(*F*_o_^2^). This list is on an absolute scale and is detwinned, merged (according to the point group of the crystal structure) and sorted, but without eliminating the anomalous contributions (except in the calculation of ϕ, so that the corresponding electron density is real). This option is essential for applying the SQUEEZE option in *PLATON* to twinned structures, but also has other uses.

RTAB D2CG followed by atom names may be used to calculate the distance between the first named atom and the unweighted centroid of the remaining atoms, together with its standard uncertainty. This can be used to calculate distances to ring centroids, for example.

As in *SHELXL97*, ‘+filename’ may be used to insert further instructions whilst reading the .ins file. These instructions are not echoed to the .res file. The new ‘++filename’ may be used to insert instructions that should be echoed to the .res file. The ‘+filename’ instruction itself is echoed to the .res file but ‘++filename’ is not. These instructions are useful for reading in long lists of restraints, *etc*.

Although the SAME instruction for generating distance restraints is very convenient, especially when combined with the use of residues (RESI) so that the same atom names may be used when there are several chemically identical solvent molecules, it is less convenient when some of those solvent molecules are disordered, for example, tetrahydrofuran (THF), with one atom either above or below the plane of the other four. A SADI instruction with no parameters now causes SADI (similar distance) restraints to be generated from all the SAME instructions. These appear after the END instruction in the .res file. They can be moved to the start of the new .ins file, and edited and extended to give fine control over the refinement of such disorders.

The TWIN instruction no longer requires integer matrix elements. The matrix is used to generate the indices of the reflections of the twin components, and if they differ by more than 0.1 from integers they are ignored. This enables the refinement of rhombohedral obverse/reverse twins, and is also useful for pseudomerohedral twins in which some of the reflections of a minor twin domain overlap nearly perfectly with reflections of the major domain and have to be taken into account, and other reflections of the minor domain do not overlap and can be ignored. If the twin components are more equal, the HKLF 5 format reflection data may be a better approach.

Details of further changes since 2008 may be found on the *SHELX* homepage (http://shelx.uni-ac.gwdg.de/SHELX/).

## Conclusions   

This account of changes and extensions to *SHELXL* since 2008 is testimony to the continuous development of the structure refinement techniques that is still taking place. In that time, CIF has advanced to become the standard for the deposition and archiving of crystallographic data, and this is reflected in many of the changes in *SHELXL*. The .ins and .hkl files used for input to *SHELXL* have remained, with very minor exceptions for which there were good reasons, upwards compatible since *SHELX76*. Another reason why *SHELX* has remained popular over many generations of computer hardware is its strict ‘no dependencies’ philosophy: no external programs, libraries (such as DLLs) or environment variables are required to run any of the *SHELX* programs (except *SHELXLE*).

## References

[bb27] Allen, F. H. (2002). *Acta Cryst.* B**58**, 380–388. 10.1107/s0108768102003890 12037359

[bb1] Berman, H. M. (2008). *Acta Cryst.* A**64**, 88–95. 10.1107/S0108767307035623 18156675

[bb2] Bruno, I. J. & Groom, C. R. (2014). *J. Comput. Aided Mol. Des.* **28**, 1015–1022. 10.1007/s10822-014-9780-9 PMC4196029 25091065

[bb3] Capelli, S. C., Bürgi, H.-B., Dittrich, B., Grabowsky, S. & Jayatilaka, D. (2014). *IUCrJ*, **1**, 361–379. 10.1107/S2052252514014845 PMC4174878 25295177

[bb4] Desiraju, G. R. & Steiner, T. (1999). *The Weak Hydrogen Bond*, pp. 350–363. Oxford University Press and IUCr.

[bb5] Diederichs, K. (2000). *J. Appl. Cryst.* **33**, 1154–1161.

[bb6] Escudero-Adán, E. C., Benet-Buchholz, J. & Ballester, P. (2014). *Acta Cryst.* B**70**, 660–668. 10.1107/S2052520614014498 25080244

[bb7] Flack, H. D. (1983). *Acta Cryst.* A**39**, 876–881.

[bb8] Gražulis, S., Daškevič, A., Merkys, A., Chateigner, D., Lutterotti, L., Quirós, M., Serebryanaya, N. R., Moeck, P., Downs, R. T. & Le Bail, A. (2012). *Nucleic Acids Res.* **40**, D420–D427. 10.1093/nar/gkr900 PMC3245043 22070882

[bb9] Gruene, T., Hahn, H. W., Luebben, A. V., Meilleur, F. & Sheldrick, G. M. (2014). *J. Appl. Cryst.* **47**, 462–466. 10.1107/S1600576713027659 PMC3937812 24587788

[bb10] Hall, S. R., Allen, F. H. & Brown, I. D. (1991). *Acta Cryst.* A**47**, 655–685.

[bb11] Hodgkin, A. L. & Keynes, R. D. (1955). *J. Physiol.* **128**, 61–88. 10.1113/jphysiol.1955.sp005291 PMC1365755 14368575

[bb12] Hooft, R. W. W., Straver, L. H. & Spek, A. L. (2008). *J. Appl. Cryst.* **41**, 96–103. 10.1107/S0021889807059870 PMC2467520 19461838

[bb13] Hübschle, C. B., Sheldrick, G. M. & Dittrich, B. (2011). *J. Appl. Cryst.* **44**, 1281–1284. 10.1107/S0021889811043202 PMC3246833 22477785

[bb14] Köpfer, D. A., Song, C., Gruene, T., Sheldrick, G. M., Zachariae, U. & de Groot, B. L. (2014). *Science*, **346**, 352–355. 10.1126/science.1254840 25324389

[bb15] Krause, L., Herbst-Irmer, R., Sheldrick, G. M. & Stalke, D. (2015). *J. Appl. Cryst.* **48**. In the press. 10.1107/S1600576714022985 PMC4453166 26089746

[bb16] Lübben, J., Volkmann, C., Grabowsky, S., Edwards, A., Morgenroth, W., Fabbiani, F. P. A., Sheldrick, G. M. & Dittrich, B. (2014). *Acta Cryst.* A**70**, 309–316. 10.1107/S2053273314010626 PMC4075069 25970187

[bb17] Parsons, S., Flack, H. D. & Wagner, T. (2013). *Acta Cryst.* B**69**, 249–259. 10.1107/S2052519213010014 PMC3661305 23719469

[bb18] Schneider, T. R. & Sheldrick, G. M. (2002). *Acta Cryst.* D**58**, 1772–1779. 10.1107/s0907444902011678 12351820

[bb19] Sheldrick, G. M. (2008). *Acta Cryst.* A**64**, 112–122. 10.1107/S0108767307043930 18156677

[bb20] Spek, A. L. (2009). *Acta Cryst.* D**65**, 148–155. 10.1107/S090744490804362X PMC2631630 19171970

[bb21] Spek, A. L. (2012). Private communication.

[bb22] Spek, A. L. (2015). *Acta Cryst.* C**71**, 9–18. 10.1107/S2053229614024929 25567569

[bb23] Taylor, R. & Kennard, O. (1982). *J. Am. Chem. Soc.* **104**, 5063–5070.

[bb24] Taylor, R. & Kennard, O. (1986). *Acta Cryst.* B**42**, 112–120.

[bb25] Thorn, A., Dittrich, B. & Sheldrick, G. M. (2012). *Acta Cryst.* A**68**, 448–451.

[bb26] Thorn, A. & Sheldrick, G. M. (2011). *J. Appl. Cryst.* **44**, 1285–1287. 10.1107/S0021889811041768 PMC3246834 22477786

